# RNA 2′-O-methylation modification and its implication in COVID-19 immunity

**DOI:** 10.1038/s41420-020-00358-z

**Published:** 2020-11-08

**Authors:** Arumugam Paramasivam

**Affiliations:** BRULAC-DRC, Saveetha Dental College & Hospital, Saveetha Institute of Medical and Technical Sciences [SIMATS], Saveetha University, Chennai, India

**Keywords:** Immune evasion, Viral infection

The recent outbreak of a novel human coronavirus infection (COVID-19), caused by severe acute respiratory syndrome coronavirus 2 (SARS-CoV-2) is a serious threat to public health, which has currently led to more than 25 million confirmed cases and more than 800 thousand deaths in 216 countries according to the World Health Organization (www.who.int). The current development of novel therapeutic and prophylactic approaches to SARS-CoV-2 infection could be categorized into at least four different strategies such as; (1) broad-spectrum antiviral agents, (2) drugs targeting the proinflammatory cytokines, (3) inhibitors of host cell proteases that participate in the priming of the viral spike protein, and (4) therapeutics targeting the virus–host interface linking the viral spike protein to the angiotensin-converting enzyme 2 (ACE2) receptor in host cells^1^. Despite significant insights into SARS-CoV-2 replication, and virus–host interactions, there is currently no approved medications or vaccines that can cure or prevent SARS-CoV-2 infection.

**Fig. 1:**
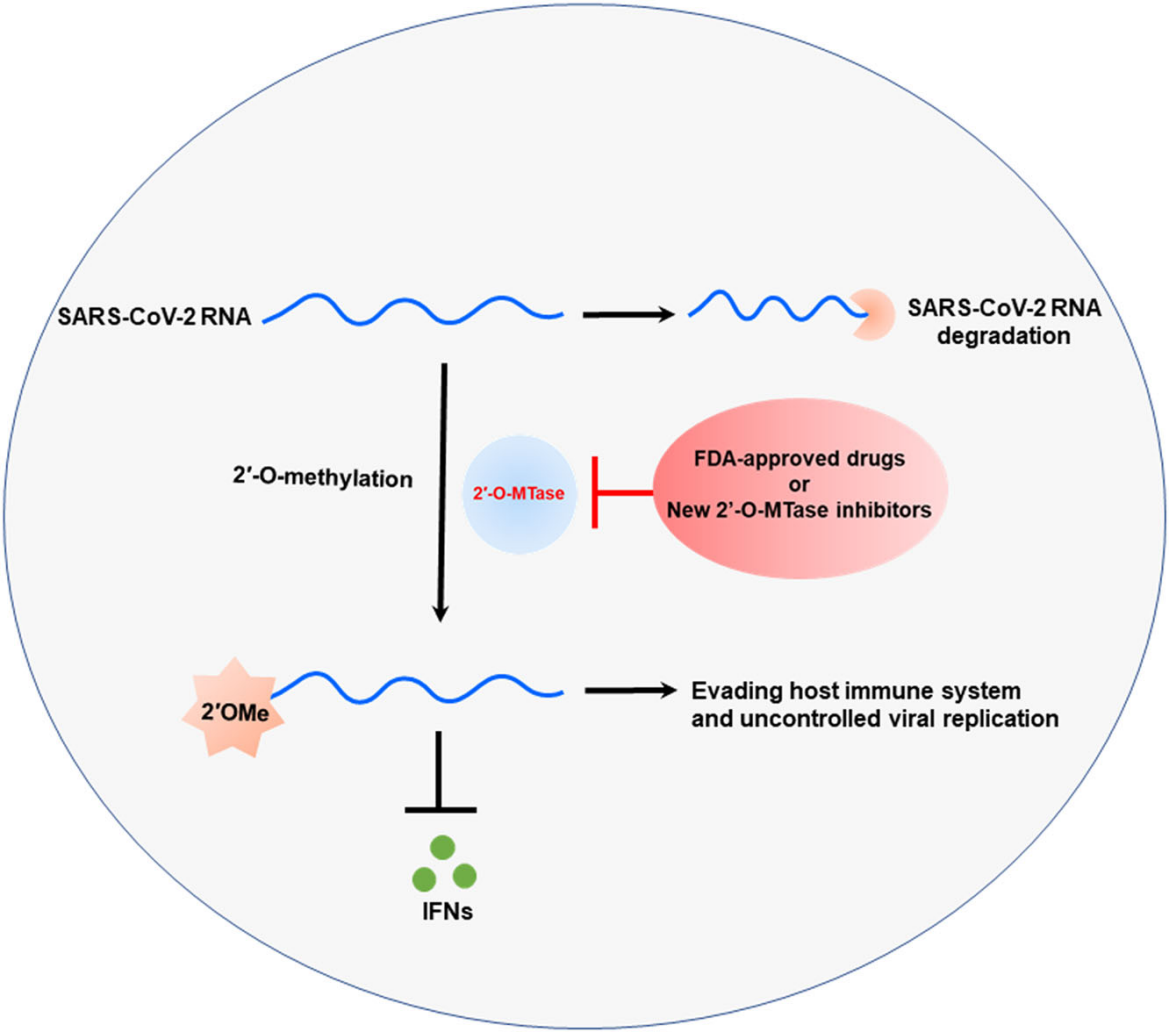
Regulation of host immune system responses by 2′-O-methyl (2′OMe) modification of SARS‐CoV‐2 RNA. SARS-CoV-2 RNA replicate in the cytoplasm of infected host cells and encode their own viral 2′-O-methyltransferase (2′-O-MTase), which catalyze the formation of 2′OMe at the 5′-end of SARS-CoV-2 RNA to impede degradation by 5′ exoribonucleases. 2′OMe modification of SARS-CoV2 RNA promotes uncontrolled replication, efficient translation, and evade recognition by the host cell innate immune system via inhibition of interferons production by immune system cells. Importantly, recent study reported that the food and drug administration (FDA) approved drugs include antivirals, alkaloids, cardiac glycosides, anticancer, and steroids act as specific inhibitor for 2′-O-MTase of SARS-CoV-2.

Coronaviruses are a group of enveloped positive-sensed RNA viruses that replicate in host cell cytoplasm through a large membrane associated RNA replication/transcription machinery comprising at least 16 virus-encoded non-structural proteins (NSP1 to NSP16). Of these, NSP16 as a viral 2′-O-methyltransferase (2′-O-MTase), which function with its co-factor NSP10 activator protein are essential for methylation of 5′-end RNA cap^2^. Recent identification of SARS-CoV-2 2′-O-MTase led to the possibility of utilizing this pathway to both attenuate SARS-CoV-2 infection and develop novel therapeutic treatment options (Fig. 1).

Viral epitranscriptomics is an emerging field, which refers to post-transcriptional modifications of RNA and plays an important role in the life cycles of different viruses including human coronavirus^3^. 2′-O-methylation (2′OMe) is one of the most common modification in the viral RNA including SARS-CoV-2 RNA^1,2^. This modification is functionally linked to all stages of RNA metabolism such as structure, stability and interactions, and plays a critical role in several biological processes such as modulating the replication of viruses and antiviral immune responses^4,5^.

Accumulating evidence indicate that 2′-O-methylation of viral RNA (2′OMe-RNA) plays an important role in evasion of cellular innate immune responses in the host cells^6,7^. Züst and colleagues recently demonstrated that 2′OMe of viral RNA contributed to evasion of the interferon (IFN)-mediated antiviral response, thereby promoting viral replication^8^. Moreover, human coronavirus mutants lacking 2′-O-MTase activity induced increased expression of IFN. These findings suggest that 2-OMe-RNA modification provides a molecular signature for discrimination of self from non-self RNA.

More recent studies demonstrated that SARS-CoV2 replicate in the cytoplasm and encode their own viral 2′-O-MTase, which catalyze the formation of cap structures at the 5′-end of SARS-CoV-2 RNA to impede degradation by 5′ exoribonucleases, ensure efficient translation, and evade recognition by the host cell innate immune system^1,2,9,10^. These studies also showed that SARS-CoV2 2′-O-MTase (NSP16 protein), which requires a cofactor NSP10 for its proper activity and the NSP10-NSP16 complex is high conserved between SARS-CoV, MERS, and SARS-CoV-2^1,2,9,10^. Interestingly, nonmethylated RNA in cytoplasm is prone to degradation and cannot be efficiently translated^9^. Crucially, the lack of 2′-O-MTase activity results in a significant attenuation of SARS-CoV infection, by decreased viral replication in vivo models^1^. Therefore, SARS-CoV2 2′-O-MTase represents a potential target for antiviral drug development and activate intrinsic cell immunity against SARS-CoV-2 infection.

Sharma and colleagues also recently identified that the SARS-CoV-2 genome encodes for 2′-O-MTase using its protein sequence, which plays an important role in methylation of viral RNA for evading host immune system. Moreover, they modeled the structure of 2′-O-MTase using a comparative modeling approach and screened the food and drug administration (FDA) approved drugs include antivirals, alkaloids, cardiac glycosides, anticancer, and steroids against 2′-O-MTase^11^. Encinar et al. also used a virtual screening approach of molecular docking of FDA approved investigational and experimental drugs to identify potential candidates that can be directed to the SARS-CoV-2 2′-O-MTase^1^. Therefore, these findings suggested that these drugs may act as specific inhibitor for SARS-CoV2 2′-O-MTase.

In conclusion, the SARS-CoV-2 genome encodes for 2′-O-MTase, which plays a key role in methylation of SARS-CoV-2 RNA for evading host immune system. Therefore, SARS-CoV2 2′-O-MTase represents a potential target for FDA-approved broad-spectrum antiviral drugs or new small molecule inhibitors development and activate intrinsic antiviral immunity against SARS-CoV-2 infection.
